# Bioinformatics Analysis to Screen the Key Prognostic Genes in Tumor Microenvironment of Bladder Cancer

**DOI:** 10.1155/2020/6034670

**Published:** 2020-02-17

**Authors:** Zhao Zhang, Dongshan Chen, Zeyan Li, Zhao Liu, Lei Yan, Zhonghua Xu

**Affiliations:** Department of Urology, Qilu Hospital of Shandong University, Wenhuaxi Road 107#, Jinan 250012, China

## Abstract

Bladder cancer (BLCA) is the fifth most common cancer and has the features of low survival rate and high morbidity and mortality. The Cancer Genome Atlas (TCGA) is a pool of global gene expression profile and contains huge amounts of cancer genomics data, which makes it possible to inquire the relationship between gene expression and prognosis of a series of malignant tumors including BLCA. Immune and stromal cells are two major components of tumor microenvironment (TME) which play an important role in judging the prognosis of tumor and influencing the progression of malignant, inflammatory, and metabolic disorders. In our study, we conducted a quantitative analysis of immune and stromal elements based on the ESTIMATE algorithm and thus divided BLCA cases into high and low groups. Then the differentially expressed genes closely related to tumor prognosis between groups were identified and had been shown to correlate with immune response and stromal alterations, which was further confirmed by functional enrichment analysis and protein-protein interaction networks. We validated those genes through BLCA dates downloaded from ArrayExpress and thus got the marker genes to predict prognosis of BLCA. Additionally, immune cell infiltration analysis explored the correlation between the verified genes and immune cells. In conclusion, we identified a series of TME-related genes that assess the prognosis and explored the interaction between TME and tumor prognosis to guide clinical individualized treatment.

## 1. Introduction

Bladder cancer is the most common malignancy of the urinary tract, and the diagnostics, treatment, and five-year survival rates for bladder cancer are largely unchanged since the 1990s [1]. Approximately 50% of those patients will have a recurrence in 2 years after an initial diagnosis and 16–25% will relapse after transurethral resection [2]. Although its exact mechanism remains obscure, many studies have shown that the tumorigenesis and progression of bladder cancer are closely related to chromosomal anomalies, epigenetic changes, and genetic polymorphism [3–5], and genetic changes are obviously involved in its initiation and prognosis [6]. Therefore, there is an urgent need to find an effective method to predict prognosis and guide clinical treatments of BLCA.

The tumor microenvironment, which is associated with tumor progression and metastasis [7, 8], is comprised of tumor cells and surroundings such as blood vessels, the extracellular matrix, and other nonmalignant cells such as tumor-associated macrophages (TAMs), mesenchymal stem/stromal cells, fibroblasts, pericytes, and immune cells [9]. Among those nonmalignant cells, stromal cells and immune cells play an important role in the whole process of tumors from happening to transferring and have definite clinical significance for diagnosis and prognosis of tumors. In the previous studies, an algorithm called ESTIMATE designed by Yoshihara et al. was used to determine the expression of certain genes of stromal cells and immune cells and calculate immune and stromal scores to infer the fraction of stromal and immune cells in tumor samples and predict the infiltration of nontumor cells [10, 11]. The previous studies have shown that the ESTIMATE algorithm based on big data is demonstrated effective in numerous cancer tissues, such as prostate cancer [12], breast cancer [13], colon cancer [14], and glioblastoma [11]. Though widely applied in varieties of cancer, prognostic evaluation of the ESTIMATE algorithm on BLCA has not yet been completely clarified. Therefore, it provides new opportunities to identify gene expression profile associated with BLCA prognosis. In our study, we took advantage of BLCA cohorts downloaded from TCGA database and ESTIMATE algorithm-derived stromal and immune scores to predict the prognosis of BLCA by a list of microenvironment-associated genes. Subsequently, another cohort of BLCA from ArrayExpress proved the prognostic value of those genes. To further elucidate related immunological mechanisms, we explored the role of the immune microenvironment in the development and prognosis of BLCA by immune cell infiltration analysis.

## 2. Materials and Methods

### 2.1. Data Source and Preprocessing

In this study, gene expression profiles of and clinical information of 412 patients with bladder cancer were acquired from the TCGA data portal (https://tcga-data.nci.nih.gov/tcga/). The ESTIMATE algorithm was used to calculate the stromal and immune scores and divided the sample patients into two high and low groups separately in accordance with the scores. In order to validate genes with prognostic significance, we downloaded a data set named E-GEOD-13507 containing microarray gene expression data associated with disease prognosis of bladder cancer from ArrayExpress (https://www.ebi.ac.uk/arrayexpress/). Tumor Immune Estimation Resource (TIMER) (https://cistrome.shinyapps.io/timer/) was used to analyze the correlation between DEGs expression and immune cell infiltration level.

### 2.2. Identification of Differentially Expressed Genes (DEGs)

According to the optimal cutoff value of immune/stromal scores through X-title software [15], we divided the patients into low and high score groups. The DEGs between low and high score groups were analyzed with package edgeR [16] in R language (version 3.5.3). The adjusted *P* value <0.05 and |log_2_FC| > 1.5 were set as the cutoff criteria.

### 2.3. Heatmaps and Clustering Analysis

The packages ggplot2 and pheatmap were used for the generation of heatmaps [17] and clustering analysis [18].

### 2.4. Function and Pathway Enrichment Analysis of DEGs

To further explore the biological processes and signal pathways of those DEGs, we performed functional analyses. Gene ontology (GO) gathers information on molecular function (MF), biological processes (BP), and cellular components (CC). Kyoto Encyclopedia of Genes and Genomes (KEGG) pathway analysis was used to excavate remarkable pathways associated with DEGs with prognostic significance. GO and KEGG were performed by R package of clusterProfiler [19]. False discovery rate (FDR) <0.05 was considered to be statistically significant.

### 2.5. Protein-Protein Interactions (PPIs) Network Construction

The DEGs were employed to construct PPIs network by Cytoscape software [20]. The related data were downloaded from STRING database [21] to construct PPI network; then hub genes were obtained. The protein product of DEGs serves as a node in the PPI network, and the interplayed protein numbers reflected the degree of interaction among proteins.

### 2.6. Survival Curves

The relationship between DEGs and survival was explored by plotting the Kaplan–Meier curve using R. The optimal cutoff value of each DEGs was determined by X-title software. *P* < 0.05 was set as the cutoff value for selecting survival-related DEGs.

## 3. Result

### 3.1. Immune Scores and Stromal Scores Are Remarkably Associated with Smoking, Race, and Bladder Tumor Staging

**Figure 1:**
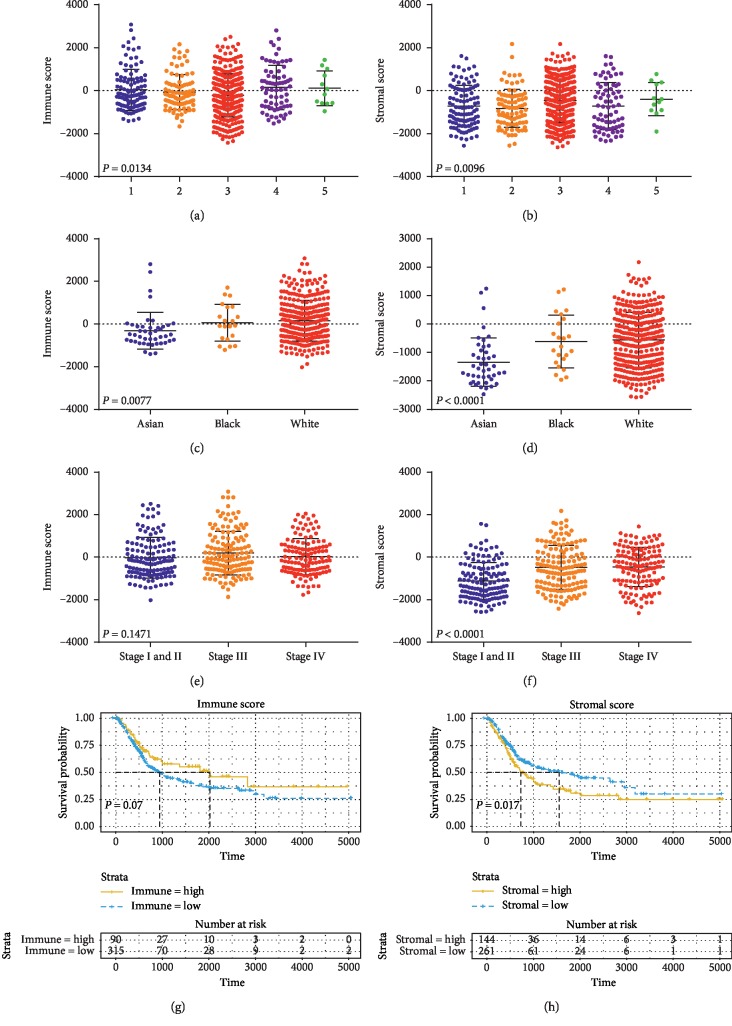
Immune scores and stromal scores are associated with smoking, race, the malignancy, and the survival probability. (A, B) Distribution of immune scores (a) and stromal scores (b) of different duration of cigarette smoking (*n* = 405, *P* < 0.05). (C, D) Distribution of immune scores (c) and stromal scores (d) for Asian, Black, and White race (*n* = 405, *P* < 0.05). (E, F) Distribution of immune scores (e) (*n* = 405, *P*=0.1471) and stromal scores (f) (*n* = 405, *P* < 0.05) of different degrees of malignancy cases. (G, H) Kaplan–Meier curves for survival probability of bladder cancer patients with low versus high immune scores (g) (*n* = 405, *P*=0.07) and stromal scores (h) (*n* = 405, *P* < 0.05).

We obtained biological omics data and clinical information of 412 patients with bladder cancer from TCGA data portal. Among those patients, 108 (26.2%) patients were female and 304 (73.8%) cases were male. Pathological data included 2 (0.5%) cases of stage I, 131 (31.8%) were stage II, 141 (34.2%) were stage III, 136 (33.0%) were stage IV, and 2 (0.5%) were not reported. On the basis of ESTIMATE algorithm, immune scores varied from −2023.05 to 3085.28, and stromal scores ranged from −2628.68 to 2175.37, respectively. The ESTIMATE algorithm provided us an opportunity to deeply probe into the complex relationship between the immune and stromal scores and the clinical characteristics. The stromal and immune scores showed a rising trend in general along with increasing duration of cigarette smoking (Figures 1(a) and 1(b)). In addition, the White race had the highest averages of immune and stromal scores, followed by that of Blacks and Asians (Figures 1(c) and 1(d)). As the stage evolved, stromal scores increased gradually (Figure 1(f)), indicating that stromal scores are useful indexes to reflect the malignancy of BLCA. However, the scatter plot shows that there is no significant correlation between the degrees of malignancy and immune scores (Figure 1(e)).

According to stromal and immune scores, we divided the BLCA cases into two halves, which are the low levels and the high level, and analyzed potential relationships between scores and survival probability. Kaplan–Meier survival curves (Figure 1(h)) showed that survival probability of cases in the low immune group was lower than the cases in the high score group, although it was not statistically significant. However, lower stromal scores groups showed higher survival probability compared to patients with higher stromal scores (Figure 1(h)).

### 3.2. Differentially Expressed Genes Were Obtained through the Comparison between High and Low Immune/Stromal Score Groups and Used for Further Analysis

**Figure 2:**
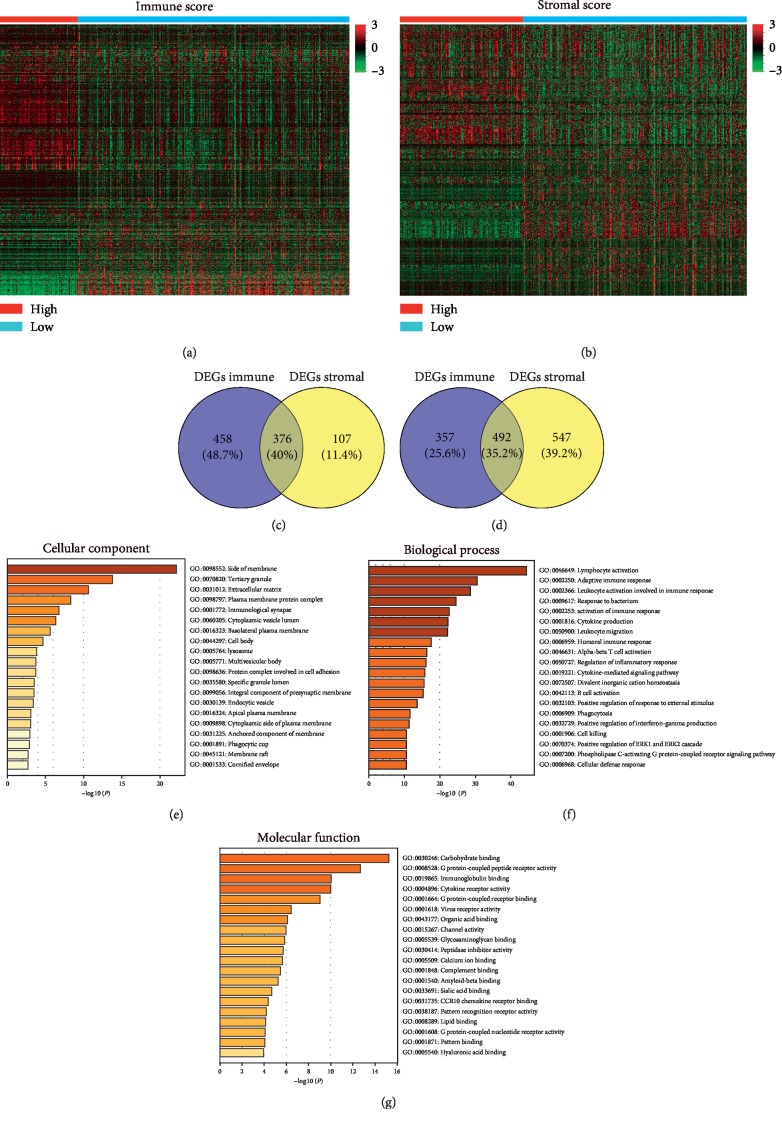
Gene expression profile is of great relevance to immune scores and stromal scores in BLCA. (a, b) Heatmaps show that differentially expressed genes profiles between high and low immune scores/stromal scores groups. Red represents higher expression genes, green represents lower expression genes, black represents same expression genes (fold change >1.5 and *P* < 0.05). (c, d) Venn diagrams show the number of coupregulated (c) or codownregulated (d) DEGs in immune and stromal score groups. (e, f, and g) The major relevant terms. *P* < 0.05.

By analyzing Affymetrix microarray data of all 412 BLCA cases downloaded from TCGA database, we obtained the differentially expressed genes (DEGs) through the consolidation and analysis of different gene expression profiles between high and low groups of immune scores and/or stromal scores. Heatmaps showed distinct gene expression profiles of cases, which belonged to high or low immune/stromal scores groups. Genes with higher expression are shown in red and those with lower expression are in green (Figures 2(a) and 2(b)). As the Venn diagrams shows, 483 genes were upregulated, and 1039 genes were downregulated on the basis of stromal scores. Likewise, 834 genes were upregulated, and 849 genes were downregulated on the basis of immune scores. The intersection of stromal and immune group was chosen for further analysis, including 376 coupregulated genes and 492 codownregulated genes (Figures 2(c) and 2(d)).

We further assessed the potential function of the DEGs by functional enrichment analysis of the co-upregulated/downregulated 868 genes. Molecular function (MF), biological processes (BP), and cellular components (CC) were explored separately based on gene ontology. And the major relevant terms include extracellular matrices, lymphocyte activation, adaptive immune response, leukocyte activation involved in immune response, immunoglobulin binding, and cytokine receptor activity, which provides further evidence that those DEGs were closely related to TME and immune response.

### 3.3. Excavation of DEGs with Prognostic Value

**Figure 3:**
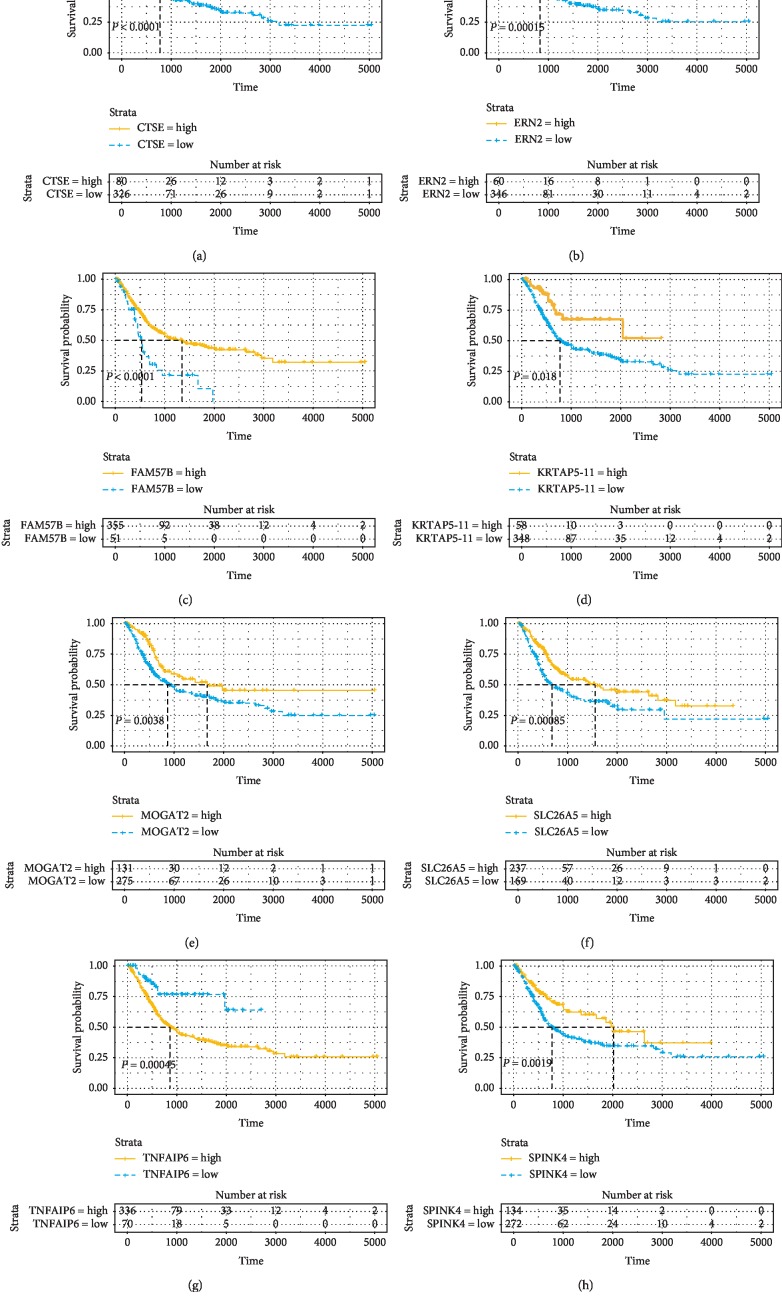
Association between DEGs expressions and overall survival in TCGA. Kaplan–Meier curves for OS (*d*) of bladder cancer patients with low versus high immune/stromal scores were made to select the DEGs. *P* < 0.05.

Based on TCGA database, we constructed survival curve to explore the correlation between DEGs and overall survival. With univariate Cox, survival analysis of 868 DEGs was performed, and 139 of them had statistical significance (*P* < 0.05). Some genes are shown in Figure 3. The expression level of some genes was positively correlated with the overall survival, while some were negative.

### 3.4. The Value of Those DEGs Related to Prognosis Was Investigated at the Level of Protein

**Figure 4:**
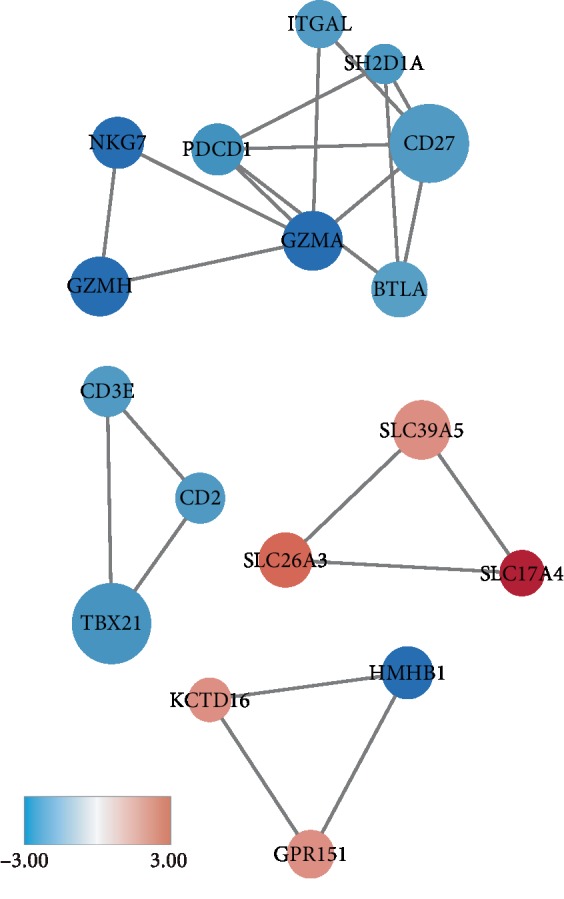
The top four remarkable PPI networks of CD27, TBX21, SLC39A5, and HMHB1 modules.

Since protein was the faithful executor of physiological function of the body, we further explored the underlying causal relationships between genes by protein-protein interaction (PPI) networks. The top four remarkable modules (Figure 4) of PPI networks, which were referred to as CD27, TBX21, SLC39A5, and HMHB1 modules for simplicity, were chosen for subsequent assessment. In the CD27 module, the nodes of CD27, PDCD1, GZMA, and SH2D1A had the closest and most extensive contacts with other members of the module. TBX21 module was made up of three immune-related genes, and SLC39A5 module contained several membrane transporter genes. For HMHB1 modules, HMHB1, as one of human minor histocompatibility antigens, had the highest degree values and played a major role in the induction of cytotoxic T lymphocyte (CTL) reactivity after allotransplantation.

### 3.5. Functional and Pathway Enrichment Analysis of DEGs Associated with Prognosis

**Figure 5:**
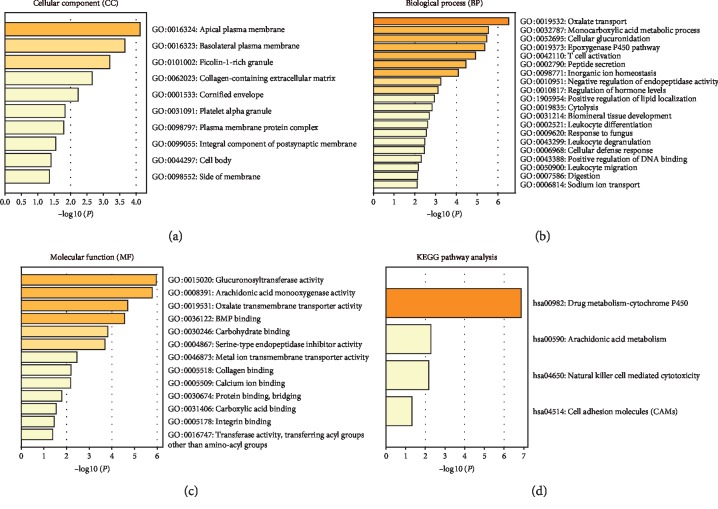
GO function analysis and KEGG pathway analysis for DEGs associated with overall survival. (a) Cellular components (CC). (b) Biological processes (BP). (c) Molecular functions (MF). (d) KEGG pathways.

In keeping with PPI network analysis, GO enrichment analysis showed that these DEGs were closely related to stromal elements and immune response. A total of 10 GO terms of cellular component, 20 GO terms of biological process, and 13 GO terms of molecular function were significantly enriched. Important GO terms included apical/basolateral plasma membrane and collagen-containing extracellular matrix (Figure 5(a)), cellular defense response and T cell activation (Figure 5(b)), and BMP binding and collagen binding (Figure 5(c)). Besides, KEGG pathway enrichment analysis was carried, and the result indicated that a set of pathways was enriched and associated with microenvironment and immune response, such as natural killer cell mediated cytotoxicity and cell adhesion molecules (CAMs).

### 3.6. Prognostic Value of DEGs Was Verified through Another BLCA Cohort from ArrayExpress Database

**Figure 6:**
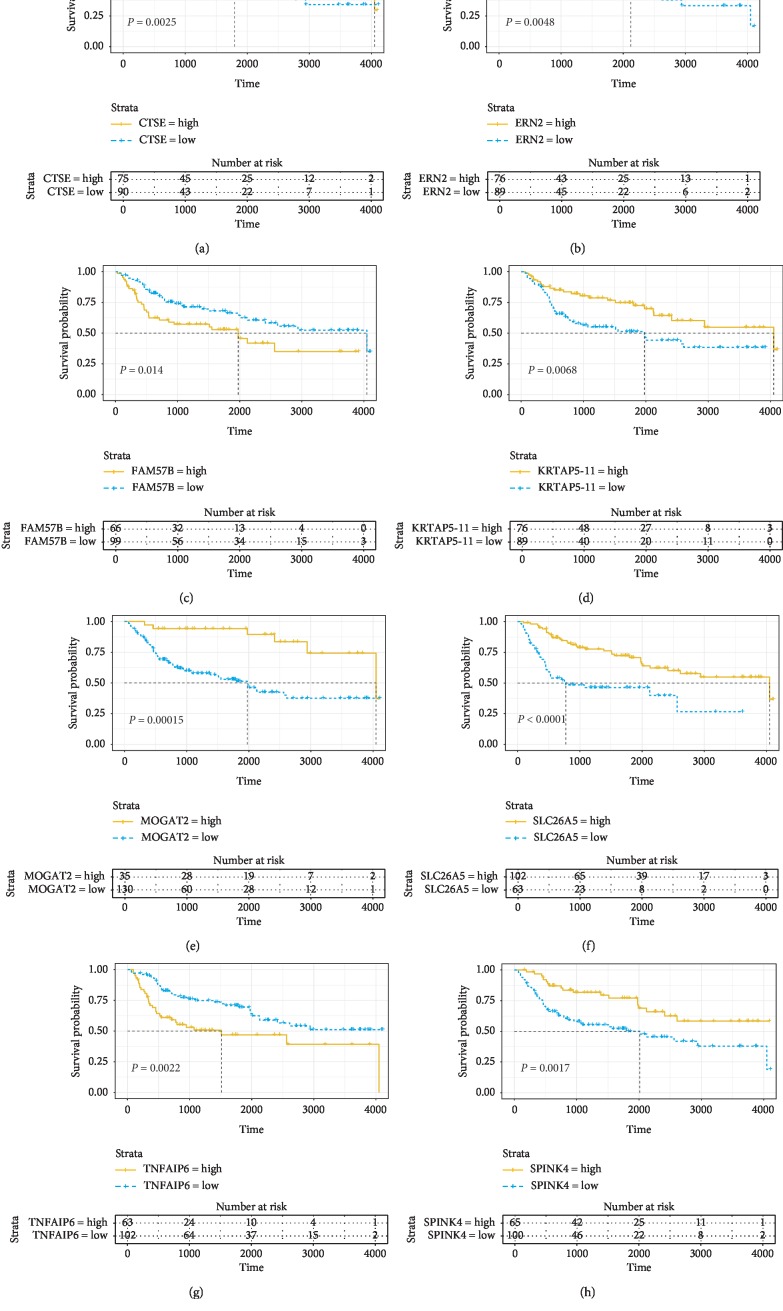
Validation of correlation of DEGs associated with prognosis extracted from TCGA database with overall survival in ArrayExpress database. Kaplan–Meier survival curves of the extracted genes from TCGA were generated to verify the prognostic significance in ArrayExpress database. *P* < 0.05.

We downloaded and analyzed a data set, named E-GEOD-13507, from ArrayExpress to see if DEGs with prognostic value were applicable for other BLCA cases. The data set was built to study the expression of prognosis-related genes and included 165 primary bladder cancer samples. Finally, a total of 14 genes had proven to be enormously valuable for predicting prognosis of BLCA. Part of the result was shown in Figure 6. The following is all genes: AADACL2, MOGAT2, COMP, KRTAP5-11, FAM57B, DSG1, TNFAIP6, SLC26A5, SPINK4, KRT1, SLC17A1, ATP12A, ERN2, and CTSE.

### 3.7. Immune Cell Infiltration Analysis Revealed the Correlations between the Identified DEGs Expression and Immunocyte

**Figure 7:**
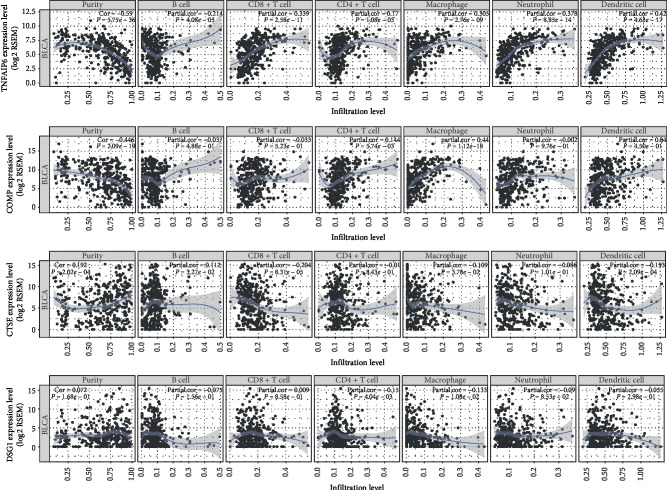
Correlation analysis between the expressions of mainly identified DEGs (TNFAIP6, CTSE, COMP, and DSG1) and infiltration levels of B cell, CD8+ T cell, CD4+ T cell, macrophage, neutrophil, and dendritic cell.

To further reveal the role of the immune microenvironment in the development and prognosis of BLCA, we analyzed the correlation between the identified DEGs and immunocyte infiltration. As Figure 7 showed, the expressions of mainly identified DEGs (TNFAIP6, CTSE, COMP, and DSG1) were separately in positive or negative relation to the infiltration level of different immune cells, indicating that the identified DEGs modulated immunologic microenvironment by influencing immune cell infiltration.

## 4. Discussion

Accumulating evidence shows that various components of TME, such as immune cells, soluble factors, and altered extracellular matrix, contribute actively to cancer progression, while linkage between TME-associated genes and cancer prognosis has not been fully elucidated. Analysis of the tumor microenvironment in patients with a variety of solid tumors has revealed that most tumor cells express antigens that can mediate recognition by host CD8+ T cells and must have evaded antitumor immune responses to grow progressively [22]. Additionally, the spontaneous T cell infiltrate of several solid tumor histologies, including breast cancer [23], renal cell carcinoma, melanoma [24], ovarian cancer, and gastrointestinal stromal tumors [25], may have significant prognostic value. Meanwhile, solid tumor stroma consists of fibroblasts, macrophages, and vascular endothelial cells, with variable amounts of extracellular matrix, all of which contribute as a support structure for tumor growth. And in addition to significantly regulating tumor growth, these components can impair host immune responses and likely contribute to the degree of immune cell infiltration [26]. Specific to BLCA, although previous findings demonstrated that some immune cells, such as CD3+ tumor-infiltrating lymphocytes (TILs), CD8+ cytotoxic T cells (CTLs), CD68+ TAM [27], and foxp+ regulatory T cells (Treg) [28], and stromal cells play a vital role in the development of BLCA, few studies have integrated multiple immunological factors into single scores to analyze the significance of immune/stroma-related genes in estimation of the prognosis of BLCA. We downloaded and analyzed microarray gene expression data from TCGA and extracted 139 prognostic DEGs, which were correlated with the stromal element and immune response. To verify whether those DEGs could be applied to other data sets, we took advantage of data from ArrayExpress, a repository to archive functional genomics data from microarray and sequencing platforms, and validated 14 genes with prognostic value in BLCA patients.

Firstly, we analyzed 868 differentially expressed genes extracted from the intersection of stromal and immune score groups. For further understanding of functions involved for the differentially expressed genes, GO analysis was performed and showed that those genes were correlated with stromal element and immune response. Growing evidences have shown that the TME not only influences the ability of growth, invasion, and transfer of tumor cells but also has profound effects on therapeutic efficacy [29]. The current study supported the conclusion that tumor stroma and immune response play a pivotal role in TME-mediated tumor progression.

Afterwards, survival analysis was carried out to demonstrate that 139 of those DEGs had the clinical value of statistically predicting survival probability. Subsequently, we made up 4 modules to comprehend protein-protein interactions and found out that those modules were associated with immune response or transporter. Furthermore, it has been confirmed that the nodes with higher degree values, such as CD27, TBX21, and HMHB1, have a stronger effect on immune cells proliferation and production and induction of cytokines [30–33]. Besides, the SLC39A5, encoding a zinc transporter, is crucial to the maintenance of TME homeostasis because zinc is a critical component of many enzymes involved in hypoxia, angiogenesis, cell proliferation, and cancer metastasis [34]. PPI analysis further strengthens the evidence linking DEGs with prognosis.

To prove the effectiveness of prognostic significance of those DEGs, we analyzed a data set from ArrayExpress, containing gene expression data of 165 primary bladder cancer samples, to see if prognostic significance of the identified DEGs is applicable for other BLCA cases and identified that 14 genes were associated with BLCA prognosis. Besides, studies in the past found that the expression of two genes (TNFAIP6 and CTSE) among those was significantly related to pathological features and strongly associated with overall survival [35, 36], suggesting that the selected genes from TCGA and ArrayExpress database based on the algorithm have important clinical value in the promotion of estimating prognosis of bladder cancer. But until now, no study has shown that the remaining 12 genes have notable correlation with BLCA prognosis and could act as a potential prognostic biomarker and therapeutic target in BLCA.

We have paid particular attention to COMP and DSG1 among the remaining genes. As a crucial component of ECM, COMP has the capacity to regulate activation of the complement system and thus innate immunity [37]. In addition, high expression of COMP protects the cells from endoplasmic reticulum (ER) stress, and cells overexpressing the COMP gene undergo a metabolic switch known as the Warburg effect [38, 39]. Furthermore, high COMP expression plays a crucial role in regulating cellular metabolism by blocking intracellular Ca^2+^ signaling and thus blocking the apoptosis process of the cells [39]. Previous studies have shown that COMP expression in breast cancer cells is significantly associated with poor prognosis [40]. Moreover, COMP expression was also found in colorectal, gastric, lung, ovarian, and pancreatic cancers as shown in the analysis of expression data using the Oncomine database [38]. DSG1, a member of the desmoglein protein subfamily [41], plays a crucial role in cell adhesion [42], whose dysfunction promotes the process of epithelial-mesenchymal transition (EMT) and thus the invasion and metastasis of cancer cells [43, 44]. It has been shown that, in a variety of tumors such as skin, head and neck, gastric, colorectal, bladder, breast, prostate, cervical, and endometrial cancers, desmosomal proteins were downregulated or even lost, which was associated with poor clinical outcome [45].

One of the basic challenges in cancer is to detect the regulators of gene expression changes in tumorigenesis and the correlation between that and prognosis. The different intracellular signal transduction pathways of low-grade and high-grade tumor heterogeneity in tumor progression and postoperative recurrence are some of the unique characteristics of bladder cancer that contribute to the challenges of evaluating the tumor prognosis [46, 47]. The Cancer Genome Atlas (TCGA) and other large-scale collaborative initiatives collect the comprehensive molecular characterization of multiple cancer types and patients' clinical data and thus provide opportunities for the studies of the genomic and molecular characterization of BLCA [48, 49].

The TME plays a vital role in tumor cell proliferation, infiltration, and metastasis and can even determine the extent of malignancy of bladder cancer [50, 51]. Therefore, the TME components determined by gene expression profiles are one of the determining factors in tumor prognosis. In this study, we integrated the gene expression data associated with extracellular matrix and immune response and obtained a great deal of DEGs with prognostic significance to improve the prediction of BLCA clinical outcomes and explore the interplay between tumor cells and the microenvironment [29, 52, 53]. Additionally, we analyzed the correlation between mainly identified DEGs (TNFAIP6, CTSE, COMP, and DSG1) and immune cell infiltration, whose result proved that the identified DEGs modulated the immune microenvironment by influencing the infiltration level of various immune cells. However, the immune-related signaling pathways and the precise mechanism of those genes affecting progression and prognosis of BLCA remained unknown. In addition to surgical treatment, adjuvant chemotherapy (ACT) is considered as a first-line regimen for advanced or metastatic urothelial bladder cancer. Previous studies have confirmed that the stromal immunotypes could serve as a practical predictive tool to identify pT3 + pT4 patients who would benefit from ACT and be used as a predictor of upcoming popularity of immunotherapy [54]. Accordingly, we could establish an independent prognostic indicator based on immune and stromal scores to analyze whether advanced or metastatic urothelial bladder cancer patients could benefit from ACT [55]. However, due to our limited level, we have not yet retrieved a data set with a sufficiently large sample size and containing ACT information. This will also be the focus of our work in the future.

## 5. Conclusions

In conclusion, we identified novel TME-related gene biomarkers using ESTIMATE algorithm based on immune and stromal scores for predicting clinical outcomes. Besides, we explored the interaction between TME and tumor prognosis, guiding the clinical individualized treatment. However, further studies should be carried out to investigate the molecular mechanisms of TME-related genes affecting prognosis.

## Data Availability

Genes data used to support the findings of this study are included within the article.
